# Risk factors for child food contamination in low‐income neighbourhoods of Maputo, Mozambique: An exploratory, cross‐sectional study

**DOI:** 10.1111/mcn.12991

**Published:** 2020-03-12

**Authors:** Sarah Bick, Lauren Perieres, Lauren D'Mello‐Guyett, Kelly K. Baker, Joe Brown, Bacelar Muneme, Rassul Nala, Robert Dreibelbis, Oliver Cumming

**Affiliations:** ^1^ Department of Disease Control London School of Hygiene and Tropical Medicine London UK; ^2^ VITROME Campus International IRD‐UCAD de l'IRD Dakar Senegal; ^3^ Department of Occupational and Environmental Health University of Iowa Iowa City Iowa USA; ^4^ School of Civil and Environmental Engineering Georgia Institute of Technology Atlanta Georgia USA; ^5^ WE Consult, Maputo Mozambique; ^6^ Ministério da Saúde Instituto Nacional de Saúde Maputo Mozambique

**Keywords:** child feeding, children, complementary foods, diarrhoea, infectious disease, low‐income countries

## Abstract

In low‐ and middle‐income countries, food may be a critical transmission route for pathogens causing childhood diarrhoea, but basic food hygiene is often overlooked in public health strategies. Characterising child food contamination and its risk factors could help prioritise interventions to reduce foodborne diarrhoeal disease, especially in low‐income urban areas where the diarrhoeal disease burden is often high. This cross‐sectional study comprised a caregiver questionnaire coupled with food sampling, and food preparation observations, among the study population of an ongoing sanitation trial in Maputo. The aim was to determine the prevalence of child food contamination and associated risk factors. The prevalence of *Enterococcus* spp., as an indicator of faecal contamination, was estimated in food samples. Risk factor analyses were performed through zero‐inflated negative binomial regression on colony counts. A modified hazard analysis and critical control point approach was used to determine critical control points (CCPs) that might effectively reduce risk. Fifty‐eight linked caregiver questionnaires and food samples were collected, and 59 food preparation observations were conducted. The prevalence of enterococci in child foods exceeding 10 colony forming units per gram was 53% (95% confidence interval [40%, 67%]). Risk factors for child food contamination were identified, including type of food, food preparation practices, and hygiene behaviours. CCPs included cooking/reheating of food and food storage and handling. This exploratory study highlights the need for more research into diarrhoeagenic pathogens and foodborne risks for children living in these challenging urban environments.

**Key messages**

- Microbial quality of child food is a major concern in low‐income unplanned urban areas, which have a high burden of diarrhoeal disease.
- In low‐income neighbourhoods of Maputo, 53% of child food samples were contaminated with faecal bacteria.
- Food contamination risk factors included preparation practices and hygiene behaviours, and potential control measures include basic measures like safe cooking, reheating and storage of food, and handwashing.
- Future research should include pathogen detection in child foods to better understand foodborne disease risk in these settings.

## 1 INTRODUCTION

In low‐ and middle‐income countries (LMICs), food may be a critical transmission route for the faecal pathogens that cause diarrhoea (Esrey, 1990). The World Health Organization (WHO) estimates that, in 2010, 230,000 deaths and 18 million disability‐adjusted life years worldwide resulted from foodborne diarrhoeal disease (World Health Organization [WHO], 2015), with the burden largely concentrated in LMICs. In these settings, faecal contamination of water (Esrey, 1990; Marino, 2007) and the environment (Curtis et al., 2011; Motarjemi, Käferstein, Moy, & Quevedo, 1993), presence of flies and rodents (Motarjemi et al., 1993), and lack of refrigeration or appropriate storage (Rowland, Barrell, & Whitehead, 1978) contribute to high levels of food contamination. Faecal bacteria can also multiply on food in hot climates (M. S. Islam, Hasan, & Khan, 1993; Kung'u et al., 2009; Zaika & Scullen, 1996), increasing the likelihood of children ingesting an infective dose at a vulnerable moment in their growth and development. High‐risk settings, such as urban “informal neighbourhoods,” where high population density and limited access to water, sanitation, and hygiene could intensify contamination (Baker et al., 2018; Cumming, Elliott, Overbo, & Bartram, 2014; Davis et al., 2018), should be a focus of food safety measures.

Children below 5 years of age are particularly susceptible to foodborne infections (Motarjemi et al., 1993). Multiple studies in LMICs have found high levels of microbial contamination in child foods (Gautam, Schmidt, Cairncross, Cavill, & Curtis, 2017; M. S. Islam et al., 2013; Touré, Coulibaly, Arby, Maiga, & Cairncross, 2011; Tsai et al., 2019) and at levels up to 10 times higher than drinking water (Black, Brown, Becker, Alim, & Merson, 1982). Diarrhoea incidence peaks at 6–11 months, in part due to introduction of new and potentially contaminated complementary foods (Bern, Martines, de Zoysa, & Glass, 1992; Fischer Walker, Perin, Aryee, Boschi‐Pinto, & Black, 2012; Motarjemi et al., 1993) and correlates with food contamination (Black et al., 1982; Ghuliani & Kaul, 1995). As children age, they may encounter additional food contamination hazards as their diets diversify (Tsai et al., 2019). Diarrhoea predisposes children to malnutrition and weakens immunity (Petri et al., 2008; Schaible & Kaufmann, 2007), causing cycles of repeated infection and deteriorating nutritional status (Baqui & Ahmed, 2006). Linked to this, growth faltering, defined by low height for age, weight for height, or weight for age, is concentrated during the first 2 years (Kotloff et al., 2013; Victora, de Onis, Hallal, Blössner, & Shrimpton, 2010).

Food hygiene has been relatively neglected in efforts to reduce this disease burden, with preventive strategies prioritising breastfeeding promotion, nutrient supplementation, and water and sanitation, each with an established evidence base (Bhutta et al., 2013; Wolf et al., 2018). A better understanding of the localised prevalence of contaminated child food in the populations most at risk would support more effective strategies (Kirk, Angulo, Havelaar, & Black, 2017). The few studies of child food hygiene in LMICs have generally focused on rural populations (Ehiri et al., 2001; Gautam et al., 2017; M. S. Islam et al., 2013; Manjang et al., 2018), with limited data from low‐income, urban neighbourhoods. Credible evidence is then needed on which risk factors should be targeted to reduce contamination in a variety of settings.

The hazard analysis and critical control point (HACCP) approach is widely used to understand potential food contamination risk and is the methodology recommended by a Food and Agriculture Organization/WHO expert committee (Food and Agriculture Organization [FAO] & WHO, 1984). This approach assesses hazards significant for food safety and identifies critical control points (CCPs) in the preparation process at which control measures might be focused to prevent, eliminate, or reduce hazards to an acceptable level (FAO & WHO Codex Alimentarius Commission, 1997). However, it lacks quantitative information on the relative impact of each CCP on food contamination that would help prioritise potential interventions.

This study applied a modified HACCP approach incorporating quantitative hazard data in low‐income unplanned neighbourhoods of Maputo, Mozambique. The aim of this study was to assess the prevalence of faecal contamination of child foods and identify associated food hygiene risk factors that might be targeted in future interventions.

## 2 METHODS

This exploratory, cross‐sectional study was nested within the ongoing MapSan trial, a controlled before‐and‐after trial measuring the health impact of an urban sanitation intervention in low‐income, informal neighbourhoods of Maputo (Brown et al., 2015). Our study's objectives were to assess the prevalence of and risk factors for child food contamination, defined as the prevalence and count of *Enterococcus* spp., a thermotolerant faecal bacterium known to be transmitted in food (Franz, Holzapfel, & Stiles, 1999) and used as an indicator of recent faecal contamination (WHO, 2017). In addition, as the study occurred after the delivery of the sanitation intervention, we assessed whether there was a difference in food contamination between compounds that had received improved, shared sanitation facilities (intervention compounds) and compounds using traditional, unimproved sanitation (control compounds).

### 2.1 Study setting and sampling

Around 70% of Maputo's inhabitants live in informal neighbourhoods covering approximately 35% of the city's area (UN‐HABITAT, 2010). These neighbourhoods include densely packed compounds, characterised by substandard housing, limited public health infrastructure, and high levels of poverty (Brown et al., 2015) and child mortality (Nhampossa et al., 2013).

Our sampling frame was drawn from the MapSan trial participant list, stratified by study arm (control/intervention). We enrolled equal numbers of eligible participants randomly from each arm. To be eligible for enrolment, participants needed to be resident in a MapSan trial compound and a caregiver of a child aged 6–60 months, with caregivers defined as the adult primarily responsible for feeding the child. Sixty‐four caregivers were enrolled, of which 58 completed a structured questionnaire and provided a food sample (29 in each trial arm) and 59 completed structured observations of child food preparation.

All data were collected between June 27 to July 24, 2018. Questionnaires and observations were recorded on tablets with forms developed using Open Data Kit. Questionnaires were written in English, translated to Portuguese and piloted in nonstudy households. All data were encrypted and kept on a secure server.

### 2.2 Caregiver questionnaire

The caregiver questionnaire addressed socio‐demographic characteristics of the household, asset ownership, compound characteristics, food preparation and feeding practices, perceptions of diarrhoeal disease risk, and whether the child had had diarrhoea in the preceding week. Potential risk factors included were predetermined prior to observations, based on food hygiene literature, and are listed in Appendix B.

To estimate relative wealth in our study population, a relative wealth index was created using principal component analysis on information on household assets and housing characteristics commonly used in the Demographic and Health Survey (MISAU/INE/INCFI, 2011). Nineteen variables were included to construct the index (Appendix A): household size, crowding, asset ownership (eight binary variables), and housing characteristics (nine variables) including water sources, building materials, and sanitation type based on MapSan trial classification, due to concordance between asset indices that include and exclude sanitation type (Rheingans, Anderson, Luyendijk, & Cumming, 2014). The first principal component was used to group households into relative wealth quintiles.

### 2.3 Food sample collection

Enumerators visited caregivers to enrol them in the study and determine when they were likely to first feed the child the following day. Enumerators returned at the designated time and asked caregivers to provide a sample of the child's meal. If no food was available, enumerators waited for it to be prepared until noon that day, or returned the next day. Enumerators administered the caregiver questionnaire alongside food sample collection. If food preparation could be observed at this visit, structured observations were conducted. Otherwise, enumerators returned to the household at a later date to observe food preparation.

Samples were collected according to standardised and pretested protocols. The following information was recorded for each sample: time of preparation, reheating, contact with other foods, and whether it had been stored in a covered container and/or refrigerated. Where the meal had multiple components, samples of all components were homogenised. Estimated 20 g of samples were placed in sterile plastic 100‐ml Whirl‐Pak bags (NASCO, Fort Atkinson, WI), up to the first line, using the same utensil used to feed the child, and temperature was taken on site. All samples were cooled, transported to the laboratory, and refrigerated at 4°C until processing less than 6 hrs after collection.

### 2.4 Structured observations

Trained enumerators conducted observations of caregivers to note ingredients and food preparation, feeding, and storage practices, using prepared guides outlining the following activities: Enumerators first asked caregivers to list the ingredients of the food that they were going to prepare and the origin and storage location of each ingredient and to briefly enumerate the steps of the preparation process; enumerators then encouraged caregivers to prepare food in their usual manner and recorded each step taken and its duration. Where possible, enumerators observed handwashing with soap (HWWS) at two moments, before food preparation and before feeding. When storage after cooking could not be observed, enumerators recorded whether it was the caregiver's intention to store the food. Observations lasted between 5 and 125 min.

### 2.5 Microbiological analysis of food samples

Enterococci were enumerated in food samples using standard membrane filtration procedures (United States Environmental Protection Agency, 2002) on *Enterococcus* indoxyl‐β‐d‐glucoside (mEI) selective medium, applied previously to measure faecal contamination (Pickering et al., 2012; Pickering, Julian, Mamuya, Boehm, & Davis, 2011). A 5‐g aliquot of each food sample taken was diluted in 50 ml of sterile water, homogenised by shaking at least 25 times and allowing to settle. The 5, 0.5, and 0.05 ml volumes of the supernatant were filtered through membranes in 50 ml of sterile water, transferred onto mEI plates, and incubated at 41°C for 20–24 hrs. Method blanks using sterilised water, positive controls using contaminated water, and replicates of one food sample were analysed each day. Colonies greater than 0.5 mm in diameter with a blue halo were identified as *Enterococcus* spp. and counted in colony forming units (CFU) per gram wet weight (CFU/g) of sample. Plates containing up to 200 colonies for the lowest dilution were counted for analysis. Counts were based upon the lowest dilution of samples except when plates for lower dilutions exceeded 200 colonies, in which case plates for the next highest dilution were counted and multiplied by the dilution factor to standardise the denominator across all samples.

### 2.6 Statistical analysis

All statistical analyses were conducted in Stata version 15.1 (StataCorp, College Station, TX, USA).

Arithmetic means were used to report colony counts (Haas, 1996). In the absence of food industry guideline values for enterococci, we interpreted results similarly to a previous food contamination assessment (Gibson, Sahanggamu, Fatmaningrum, Curtis, & White, 2017) and coliform guidelines used in the milk industry (U.S. Department of Health and Human Services, Public Health Service, & Food and Drug Administration, 2009), defining an outcome of >10 CFU/g as “contaminated.” We calculated the prevalence of *Enterococcus* spp. contamination (>10 CFU/g) in child foods alongside 95% confidence intervals (CIs).

Exposures of interest were caregiver demographics, including wealth and education; factors related to environmental contamination, including water source and treatment and sanitation type; HWWS; and type and preparation of food. The ratio of household members to rooms was used as a proxy measure for crowding. All variables were converted to categorical variables based on appropriate threshold values, and food types were grouped based on common ingredients of samples. These exposures were then cross‐tabulated with the binary contamination outcome (>10 CFU/g) and tested for crude associations using Fisher's exact test.

For regression analyses, counts lower than 10 CFU/g were coded as below safe limits. Categorical variables were ordinal, apart from food type, for which each food category was treated as an individual dummy variable. Overdispersion and excessive zeros (27/58) indicated appropriate use of a zero‐inflated negative binomial regression model. Due to the relatively small sample size for food samples (*n* = 58), nonparametric bootstrapping as described by Efron (1979) was applied to subsequent risk factor analyses.

Variables with a *p* value below .2 in Fisher's exact test, as well as those variables that perfectly predicted the outcome, were considered for inclusion in the logistic portion of the model. Variables were tested for associations with bacterial count using bivariate negative binomial regression, and variables with a *p* value below .2 were considered for inclusion in the negative binomial portion of the multivariable model. For each iteration of the model, each variable was added individually to the logistic portion. Akaike information criterion (AIC) was used to compare the base model with each new model, with the variable retained where this statistic was minimised. This process was repeated until no variables improved model fit. Variables were then added to the negative binomial portion in the same manner. Variables excluded at the screening stage were checked to determine if their inclusion in either portion of the model improved the fit; none met this criterion. Risk factors included in the final multivariable model were checked for multicollinearity, and variables exceeding a variance inflation factor of 10 were removed. The final zero‐inflated negative binomial model was compared with negative binomial, Poisson, and zero‐inflated Poisson regression models using AIC and likelihood ratio tests. There was very strong evidence supporting the preference of the zero‐inflated negative binomial model (AIC: 505) over Poisson (AIC: 44,400) and zero‐inflated Poisson (AIC: 42,700) models, and weak evidence preferring it over a negative binomial model (AIC: 523), on the basis of these model fit statistics.

### 2.7 HACCP analysis methodology

HACCP analyses typically include collection of detailed information on the food product (composition, pH, A_W_, etc.), full assessment of physical, chemical and biological hazards at each stage in the operation, and specification of critical limits (e.g., time and temperature criteria) and monitoring procedures for each CCP. A range of microbiological organisms of concern and associated toxins would also be considered (FAO & WHO Codex Alimentarius Commission, 1997). Logistical constraints for this small, exploratory study led to modification of the standard HACCP to assess microbiological hazards using an indicator for the likely presence of faecal pathogens, restricted to prepared foods at the point of feeding as the critical time for the child's safety, and incorporated quantitative risk factor analysis to deduce the relative importance of intermediate steps.

We analysed structured observation data and created food preparation flow diagrams for each food type observed, with foods then grouped into categories based on ingredients and preparation processes, as well as a general food‐flow diagram for all foods. We identified hazards by prevalence and magnitude of contamination by *Enterococcus* spp. in child foods. We considered the steps observed in the food preparation process and used risk factors identified from multivariable analysis to identify a range of points in the process where a step might increase, or a control measure might reduce, the prevalence or magnitude of contamination. We also compared frequencies of these significant risk factors in observations and used temperature measurements at time of feeding to assess the extent of reheating. We then identified CCPs out of the range of control points based on whether (a) preventive control measures can be applied, (b) loss of control could increase contamination to unacceptable levels, and (c) no subsequent steps exist to control the hazard (NSF (National Sanitation Foundation), 2006). We annotated the food‐flow diagrams with identified CCPs and proposed feasible control measures to reduce risk of faecal contamination of child foods in this setting.

### 2.8 Ethical considerations

Ethical clearance was obtained prior to enrolment of study participants from the LSHTM (LSHTM MSc Ethics Ref: 15336 and 15112) and the National Bio‐Ethics Committee of Mozambique (CNBS), as an amendment to the MapSan trial protocol (Reference: 204/CNBS/18). Informed written consent was obtained from all study participants. No incentives were given prior to participation, but households were reimbursed for food samples with a bag of rice following data collection. Personal identifiers were removed from all data records and excluded from analysis.

## 3 RESULTS

### 3.1 Characteristics of food samples

**Figure 1:**
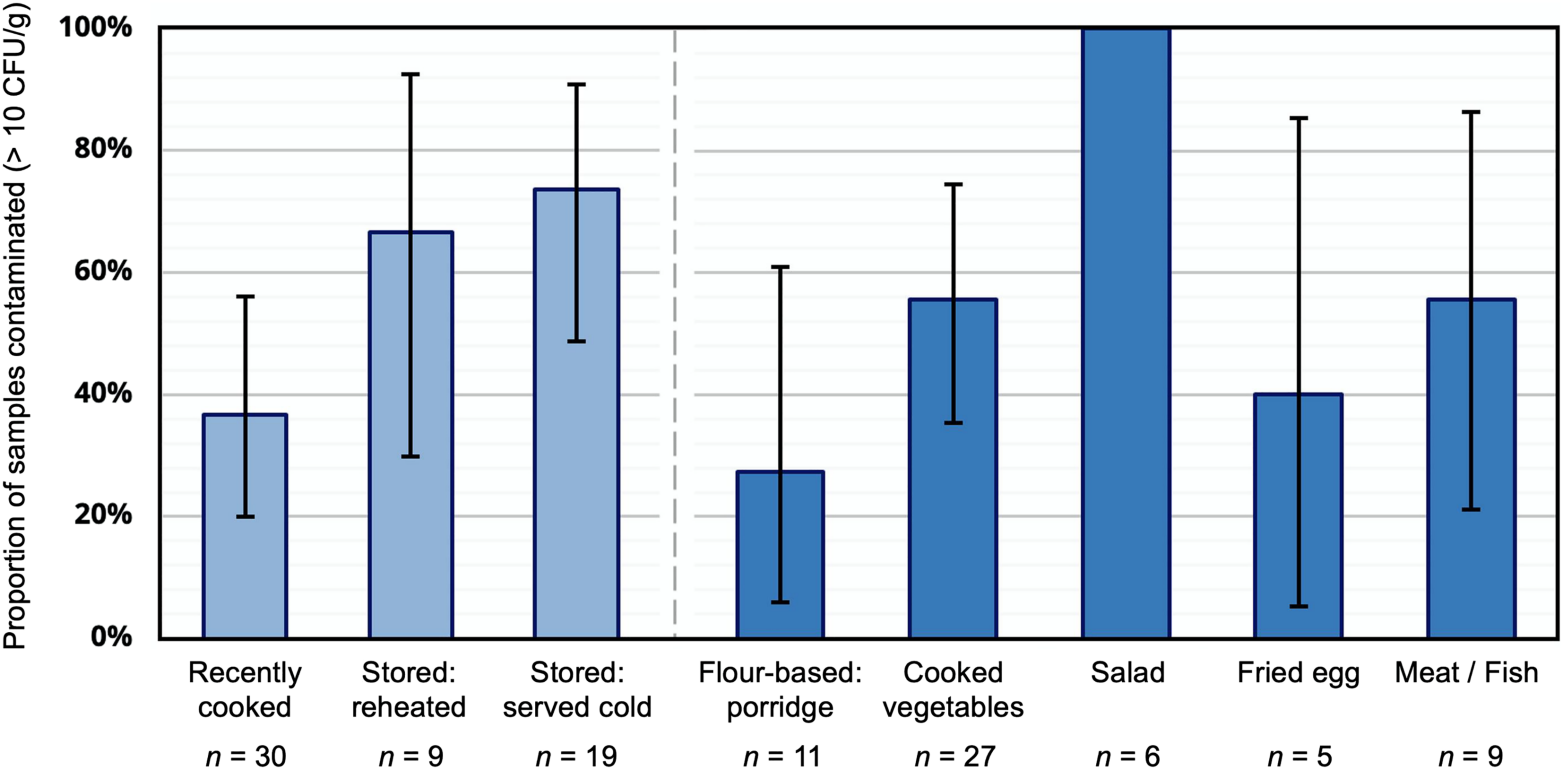
Proportion of child food samples contaminated with faecal bacteria (>10 colony forming units [CFU] per gram [CFU/g]) in Maputo, Mozambique, by heating practice (recently cooked, stored and reheated, or stored and served cold) and food type

The child foods sampled for microbiological analysis were grouped into five categories: (a) boiled cornmeal porridge (*n* = 11), (b) cooked vegetables (e.g., cabbage) served with rice, pasta, or *xima*, cornmeal boiled to a firm dough‐like consistency (*n* = 27), (c) salad (*n* = 6), (d) fried egg (*n* = 5), and (e) meat/fish (*n* = 9). Of 58 food samples analysed, 31 samples exceeded 10 CFU/g *Enterococcus* spp. (53%, 95% CI [40%, 67%]). Among these, the mean enterococci count was 854 CFU/g. Counts spanned three orders of magnitude up to 8,100 CFU/g for the most heavily contaminated sample. Proportion of samples contaminated varied across food types and by storage practices (Figure 1). All salads were contaminated, and cooked vegetables and meat/fish were often contaminated (both 56%). Food preparation factors are described in detail later.

### 3.2 Characteristics of study participants and exposures

A full list of potential risk factors cross‐tabulated with the binary contamination outcome are presented in Appendix B. Fourteen exposures had a *p* value below .2 or perfectly predicted food contamination above safe limits (Table 1) and were considered for inclusion in the logistic portion of the multivariable regression model. The same risk factors were tested for associations with the bacterial count using bivariate negative binomial regression modelling (Appendix C). The seven variables with a *p* value below .2 (Table 1) were considered for inclusion in the negative binomial portion of the multivariable model.

**Table 1:** Sixteen risk factors considered for inclusion in the multivariable regression model of food contamination, out of characteristics of 58 caregivers and their households in Maputo, Mozambique

Risk factor	*N* (%)	Contaminated (%)	Fisher's exact test	Negative binomial regression	
			*p* value	Coefficient^a^	*p* value
Caregiver sex					
Female	57 (98)	31 (54)	.47^b^		
Male	1 (2)	0 (0)			
No. of household members					
2–4	27 (47)	11 (41)	.11	Ref	
5+	31 (53)	20 (65)		2.5	<.01
No. of children under 5 years					
1	37 (64)	17 (46)	.17		
2+	21 (36)	14 (67)			
Wealth quintile					
1st	12 (21)	8 (67)		Ref	
2nd	11 (19)	5 (45)		−2.8	<.01
3rd	12 (21)	7 (58)		0.11	.94
4th	11 (19)	6 (55)		−0.63	.67
5th	12 (21)	5 (42)		−0.56	.70
Child faeces observed on premises					
Yes	2 (3)	2 (100)	.49^b^		
No	56 (97)	29 (52)			
Soap availability					
Yes	53 (91)	26 (49)	.06^b^		
No	5 (9)	5 (100)			
Self‐reported HWWS					
Yes	35 (60)	16 (46)	.18		
No	23 (40)	15 (65)			
HWWS after changing nappies					
Yes	3 (5)	0 (0)	.10^b^	−26	<.01
No	55 (95)	31 (56)		Ref	
HWWS before preparing food					
Yes	22 (38)	11 (50)		1.3	.14
No	36 (62)	20 (56)		Ref	
HWWS before eating					
Yes	30 (52)	13 (43)	.12		
No	28 (48)	18 (64)			
HWWS before feeding the child					
Yes	16 (28)	6 (38)	.15		
No	42 (72)	25 (60)			
Main ingredients (ref: Ingredient not included)					
Flour‐based: Porridge	11 (19)	3 (27)	.06	−4.2	<.01
Cooked vegetables	27 (47)	15 (56)		1.2	.12
Salad	6 (10)	6 (100)			
Fried egg	5 (9)	2 (40)			
Meat/fish	9 (16)	5 (56)			
Contact with other foods (same container/utensil)					
Yes	23 (40)	15 (65)	.18		
No	35 (60)	16 (46)			
Storage after cooking^c^					
Recently cooked	30 (58)	11 (37)	.09	Ref	
Cooked earlier, stored	22 (42)	14 (64)		1.3	.09
Food stored in covered container^d^					
Yes	16 (73)	8 (50)	.05^b^		
No	6 (27)	6 (100)			
Food refrigerated^d^					
Yes	1 (5)	0 (0)	.36^b^	−27	<.01
No	21 (95)	14 (67)		Ref	

Abbreviation: HWWS, handwashing with soap.

a Coefficient relates to the increase in log count of enterococci.

b Perfectly predicts the outcome.

c Out of cooked food (excluding salad).

d Out of food stored after cooking.

Caregivers reporting responsibility for feeding the child were predominantly female (98%) and often the child's mother (74%). The mean caregiver age was 34 years (range 17–79 years), and most (86%) had at least primary education. The mean child age was 31 months (range 7–56 months). Ten percent of children in the sample had diarrhoea in the week preceding (95% CI [4, 21]). Ninety‐five percent of children in the study had been breastfed at some point, but only 5% were still breastfed at the time of the study. The median number of household members was six (range 2–13), and houses were crowded—52% of households had more than four members per room. There was weak evidence that households with more than one child under 5 years had more contaminated food samples (67% vs. 46%, *p* = .17). Prevalence of food contamination did not vary by relative wealth quintile (*p* = .46).

Soap was present in 91% of households, and 60% reported HWWS. All households without soap had contaminated food samples, and there was weak evidence that caregivers reporting HWWS had fewer contaminated food samples (46% vs. 65%, *p* = .18). Key moments caregivers reported HWWS were after defecation (47%), before eating (52%), and before preparing food (38%). The rarest mentioned moment, after changing nappies, perfectly predicted no contamination.

Respondents took water from a tap in the compound (57%) and stored it in a container with a closed lid (84%). Ten caregivers (17%) reported treating water, seven of these by boiling. There was no difference in proportion of samples contaminated by water source, storage or treatment, or between intervention and control arms of the MapSan trial (55% vs. 52%, *p* = 1.00). Children's faeces were observed in two compounds (3%) and perfectly predicted contamination. Animals were observed in 39 compounds (67%).

Most caregivers (74%) prepared food inside their home. Fifty‐eight percent of cooked food samples, taken at time of feeding, were taken immediately after cooking, and others taken after the food had been stored, and later reheated (17%) or given cold (25%). Porridge and fried egg were served immediately and never stored. Salad was the only food not cooked. Prevalence of contamination varied by storage after cooking (64% vs. 37%, *p* = .05), but not by subsequent reheating (67% vs. 62% served cold, *p* = .81). Mean temperature of food samples at time of feeding was 37°C in reheated foods and 45°C for those recently cooked, with weak evidence of a temperature difference (*p* = .08).

Because samples were taken in the morning, foods not recently cooked had been stored for over 11 hrs overnight (mean: 20 hrs; 95% CI [18, 23]). 73% of stored foods had been kept in a covered container. Although 26% of study households used a refrigerator/freezer to store raw ingredients, only one food sample had been refrigerated. Of food stored after cooking, covered storage and refrigeration perfectly predicted contamination.

### 3.3 Multivariable regression

The multivariable regression model comprised nine risk factors for child food contamination (Table 2), in two distinct sets: five associated with the odds of food samples being below safe limits (<10 CFU/g) or nonzero; and four associated with increased or decreased bacterial count in nonzero samples. For the logistic portion of the model, while the small sample size (*n* = 58) with several perfect predictors for the binary contamination outcome produced some extreme estimates, there was strong evidence that soap availability and HWWS after changing nappies were associated with increased log odds of excess zeros in samples (20 and 21 log [odds] increase, respectively; *p* < .01). Salad and food stored after cooking were associated with reduced odds of zero counts (21 and 22 log [odds] reduction, respectively; *p* < .01). For nonzeros, there was evidence of lower counts for porridge (3.2 log [count] reduction, *p* = .10) and for stored foods being refrigerated (25 log [count] reduction, *p* < .01). The two sets approximate to those that may affect pathogen presence or survival—including hygiene behaviours and cooking food to boiling temperatures—and those that affect bacterial proliferation—including safe storage and type of food that facilitated growth.

**Table 2:** Multivariable zero‐inflated negative binomial regression model comprising nine risk factors for child food contamination, out of characteristics of 58 caregivers and their households in Maputo, Mozambique

	Logistic portion			
Risk factor	Coefficient^a^	*z*	95% CI	*p* value
Soap available—Yes (ref: No)	20	20	[18, 22]	<.01
HWWS after cleaning nappies—Yes (ref: No)	21	16	[18, 24]	<.01
Type of food—Salad (ref: Not salad)	−21	−15	[−24, −18]	<.01
Food stored after cooking (ref: Cooked and not stored; not calculated: Uncooked food)	−22	−20	[−24, −19]	<.01
Food stored in covered container (ref: Stored uncovered; not calculated: Not stored after cooking)	20	20	[18, 23]	<.01
Constant	−20	−22	[−22, −18]	<.01

	Negative binomial portion			
Risk factors	Coefficient^b^	*z*	95% CI	*p* value
Type of food—Porridge (ref: Not porridge)	−3.2	−1.7	[−6.9, 0.59]	.10
Type of food—Cooked vegetables (ref: Not cooked vegetables)	1.1	1.5	[−0.36, 2.5]	.14
Food refrigerated (ref: Stored at ambient temperature;not calculated: Not stored)	−25	−3.0	[−42, −8.8]	<.01
Wealth quintile—2nd (ref: 1st quintile; not calculated: 3rd, 4th, 5th quintile)	−2.7	−1.1	[−7.6, 2.1]	.27
Constant	6.4	12	[5.3, 7.4]	<.01

Abbreviations: CI, confidence interval; HWWS, handwashing with soap.

a Coefficient relates to the increase in log odds of excess zeros.

b Coefficient relates to the increase in log count.

### 3.4 Structured observations and HACCP analysis

**Figure 2:**
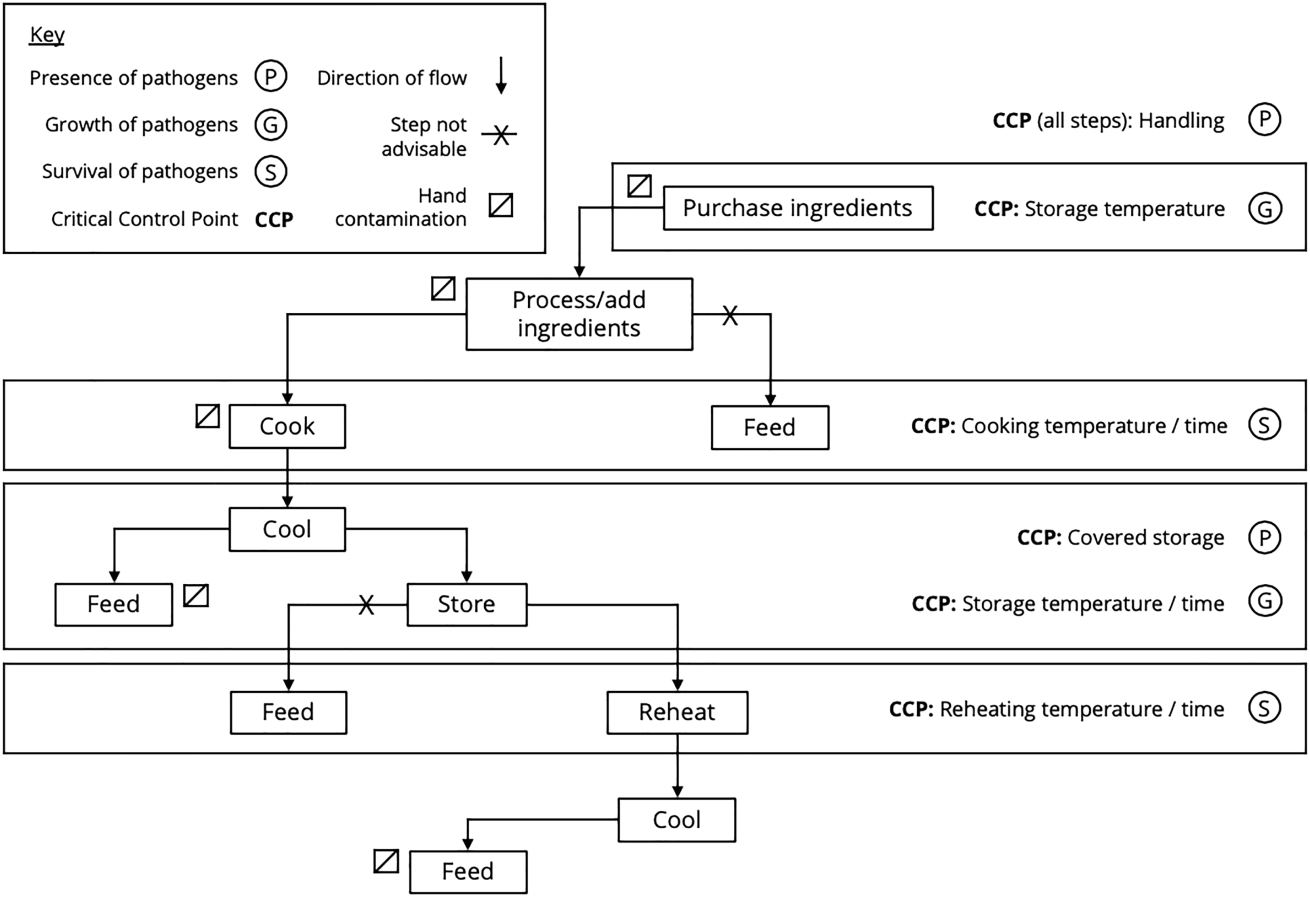
Flow chart of key steps in the preparation of child foods in Maputo, Mozambique, and associated critical control points (CCPs)

We observed caregivers preparing children's food and recorded frequency of the factors identified in the risk factor analyses (Table 3), alongside information on storage and processing of ingredients. Food‐specific CCPs and control measures are reported. The child foods observed were grouped into five categories: (a) flour‐based food (porridge and *xima*) (*n* = 20), (b) cooked vegetables with rice or pasta (*n* = 21), (c) salad (*n* = 7), (d) fried egg (*n* = 6), and (e) meat/fish (*n* = 5); *xima* was grouped with porridge due to similar preparation processes. A general food preparation flow diagram is provided in Figure 2, and food preparation flow diagrams were created for each food category in Appendix D.

**Table 3:** Observed preparation practices, critical control points, and control measures for common child foods in Maputo, Mozambique

Observed risks	Flour‐based: Porridge and *xima* (*n* = 20)	Cooked vegetables with rice/pasta (*n* = 21)	Salad (*n* = 7)	Fried egg (*n* = 6)	Meat/fish (*n* = 5)
Storage/processing of ingredients					
Not covered		15/16 (94%)	7/7 (100%)	6/6 (100%)	
Not refrigerated		15/16 (94%)	7/7 (100%)		3/5 (60%)
Not washed		8/17 (47%)	0/6 (0%)		
Handling					
No HWWS before food preparation	11/14 (79%)	12/13 (92%)	5/7 (71%)	3/4 (75%)	2/3 (67%)
No HWWS before feeding	12/18 (67%)	10/14 (71%)	5/7 (71%)	2/5 (40%)	1/4 (25%)
Cooking					
Not cooked to boiling	0/20 (0%)	0/21 (0%)	7/7 (100%)	0/6 (0%)	0/5 (0%)
Storage after feeding					
Intend to store food	2/12 (17%)	12/17 (71%)	1/5 (20%)	2/4 (50%)	2/4 (50%)
Not covered	2/2 (100%)	4/12 (33%)	1/1 (100%)	2/2 (100%)	0/2 (0%)
Not refrigerated	2/2 (100%)	11/12 (92%)	1/1 (100%)	2/2 (100%)	2/2 (100%)
Not reheated	1/2 (50%)	4/6 (67%)	1/1 (100%)	2/2 (100%)	0/2 (0%)

Critical control points (CCPs)					
	• Handling • Covered storage (*xima*) • Storage temperature, time (*xima*) • Reheating temp., time (*xima*)	• Handling • Covered storage • Storage temp., time • Reheating temperature, time	• Receipt—Vendor handling • Handling • Covered storage • Storage temperature • No cooking (temperature, time)	• Handling • Covered storage • Storage temp., time • Reheating temperature, time	• Handling • Covered storage • Storage temperature, time • Reheating temp., time

Control measures					
	• HWWS • Avoid storing for long periods (*xima*) • Store covered, refrigerate where possible (*xima*) • Reheat thoroughly (*xima*)	• HWWS • Avoid storing for long periods • Store covered, refrigerate where possible • Reheat thoroughly	• Avoid these foods—No cooking • HWWS • Store covered, refrigerate where possible	• HWWS • Avoid storing for long periods • Store covered, refrigerate where possible • Reheat thoroughly	• HWWS • Avoid storing for long periods • Refrigerate where possible • Reheat thoroughly

*Note.* Denominators are number of recorded events.

Abbreviation: HWWS, handwashing with soap.

For all food categories, HWWS was rare before food preparation (20%) and feeding (38%). Additional ingredients that may have been contaminated, such as bread or rice, were frequently added before feeding. Many caregivers (45%) reported intending to store the food after cooking. However, most children were fed promptly following food preparation, and all cooked foods were boiled. Only two flour‐based foods, both *xima,* were stored, but most cooked vegetables (71%) were stored after cooking. Only meat/fish or cooked vegetables were observed to be covered during storage and reheated. For meat/fish, two out of five caregivers stored raw ingredients in a refrigerator/freezer, but no caregivers were observed refrigerating food after cooking. For salads, caregivers washed raw vegetables thoroughly using water collected from a tap in the compound and stored in the home; this water was often not covered (43%) and not treated (100%) before use.

Observations and quantitative risk factor data were combined to produce a general food‐flow diagram with six CCPs relevant for all categories of child food preparation (Figure 2) and the following control measures:

1. *Cook food thoroughly*, as evidenced by the large decrease in log odds of excess zeros in salads despite washing, and no subsequent steps to control hazards.
2. *Avoid storing food for long periods.* Instead, *feed after cooking* where feasible. Storage after cooking was associated with nonzero counts, and porridge, which is never stored, was associated with lower counts.
3. If food is stored, *keep it in a covered container*, due to association with zero counts, and *refrigerated where possible*, because of the large reduction in bacterial count.
4. Similarly, *refrigerate ingredients* (meat/fish and vegetables) where possible. This practice was rarely observed despite refrigeration being available.
5. *Reheat food thoroughly after storage*, to boiling temperatures. Although not observed, temperatures at feeding suggested inadequacy of this practice, and no subsequent step exists to control hazards.
6. *HWWS at key moments* as a general control method. Those specific moments could not be separated, yet soap availability and HWWS after changing nappies were associated with increased odds of excess zeros, and HWWS was rarely observed.

## 4 DISCUSSION

This study assessed child food contamination in low‐income, informal, and densely populated neighbourhoods of Maputo, Mozambique; hazard analysis data to date have been limited for such settings. We used the HACCP approach to assess prevalence of child food contamination in this setting and provide new information about unsafe food hygiene behaviours that contribute to contamination. Fifty‐three percent of food samples in our study were contaminated above safe limits. Risk factors for contamination included HWWS, cooking, and storage practices. Six CCPs were identified, including cooking/reheating, handling and storage, and specific factors examined in relation to existing literature and best practice.

The 53% prevalence of contamination by *Enterococcus* spp. (>10 CFU/g) was substantial in our study population and matches the 40–58% frequencies of faecal indicator bacteria that have been well‐documented in child foods in other LMICs (Ghuliani & Kaul, 1995; Gibson et al., 2017; Gil et al., 2014; M. A. Islam et al., 2012; Parvez et al., 2017), including in low‐income urban/peri‐urban areas (Black et al., 1989; Mølbak, Højlyng, Jepsen, & Gaarslev, 1989; Sheth, Patel, Sharma, & Seshadri, 2000; Touré et al., 2011). Though concordance between indicator bacteria and enteric pathogens in food is poorly understood, high pathogen prevalence in food in Kenya (Tsai et al., 2019) raises concern that enteric pathogens may occur at higher than acceptable rates in child foods in this population.

The risk factors we identified were largely consistent with other quantitative food hygiene studies (Afifi, Nasser, Shalaby, & Atlam, 1998; Henry, Patwary, Huttly, & Aziz, 1990; Imong et al., 1995; Kung'u et al., 2009; Parvez et al., 2017), with some differences. For example, the protective effect of covering food during storage as seen in our study is similar to findings in Egypt (Afifi et al., 1998) and Bangladesh (Parvez et al., 2017), but not other findings in Bangladesh (Henry et al., 1990) or Thailand (Imong et al., 1995). The corrective measures proposed correspond with previous HACCP analyses in LMICs used to develop food hygiene interventions (Gautam et al., 2017; M. S. Islam et al., 2013; Manjang et al., 2018; Touré, Coulibaly, Arby, Maiga, & Cairncross, 2013), all implementing HWWS by caregivers before food preparation and feeding, and thorough reheating of cooked food.

Some safe practices were observed: All foods cooked under observation were boiled, utensils were visibly clean, and children were fed promptly after cooking. Control measures identified broadly match existing best practice, as seen in the WHO “Five Keys to Safer Food” message: (a) Keep clean, (b) separate raw and cooked, (c) cook thoroughly, (d) keep food at safe temperature, and (e) use safe water and raw materials (WHO, 2006), and therefore many hazards may be mitigated by guideline practices.

Fresh produce was associated with greater risk of contamination than cooked foods, and made up 12% of observations. In previous studies, 30–80% of raw food has been found to harbour enteric pathogens (Lanata, 2003). In our observations, ingredients were mostly bought from the market, the *banca* or *contentor*, street stores where foods are exposed, presenting potential risk. Porridge, which is cooked to high temperatures in small batches, had a significantly lower microbial load. Cooking to a minimum of 70°C is recommended (WHO, 1996). Temperatures of food samples at time of feeding support evidence that reheating may often not reach cooking temperatures (Schmitt et al., 1997), and prevalence of contamination did not vary after reheating. Often lack of fuel can be a barrier to safe cooking practices, for which suitable solutions will have to be assessed.

The higher prevalence of contamination among food stored overnight confirms previous observations of bacterial proliferation in stored food in hot climates (Zaika & Scullen, 1996). Bacteria grow exponentially at such optimal temperatures (20–40°C) and plateau, favouring toxin formation (Motarjemi & Schothorst, 1999). It is therefore recommended that child foods not be stored after first feeding. Often, time constraints govern feeding practices; food samples were 3.7 times more likely to have been stored after cooking for each additional child under 5 years in the household (*p* = .04). Number of children under the age of 5 years in the household may have been associated with contamination due to practicalities of cooking in bulk. If foods must be kept, using a covered container and refrigeration were highlighted as safe practices.

Although even washed utensils and containers have shown high counts of faecal bacteria in other studies (Barrell & Rowland, 1980), no associations between cleaning of utensils or surfaces and food contamination were found in this study. Presence of children's faeces in the vicinity and HWWS after changing nappies appear to affect child food contamination; however, none of the caregivers mentioned children's faeces as a cause of diarrhoea. Low perception of children's stools being contaminating has been associated with contaminated food (Bukenya, Kaser, & Nwokolo, 1990), potentially reducing effects of caregivers reporting what is considered “good behaviour.” HWWS before food preparation and feeding was rarely observed, corroborating findings in Nigeria (Ehiri et al., 2001) and India (Sheth et al., 2000). Faecal coliforms have been detected on 14–79% of mothers' hands in LMICs (Lanata, 2003). Although presence of soap does not guarantee its appropriate use (Ram, 2013), its association with microbial load lends support to HWWS as a critical food hygiene behaviour.

The lack of a significant difference in food contamination between intervention and control arms of the MapSan trial (Brown et al., 2015)—and therefore despite access to improved, shared sanitation—is notable; the mode of exposure of child foods to enteric pathogens may operate largely independently to environmental conditions. Limited access to sanitation and safe water is frequently associated with food contamination (Motarjemi et al., 1993), and recent studies implicate domestic animals as a potential source of contamination (Barnes, Anderson, Mumma, Mahmud, & Cumming, 2018; Ercumen et al., 2017). We found no associations with domestic animals or treatment or storage of water, although microbial content or quantity of water were not explored.

### 4.1 Limitations

As this was an exploratory, cross‐sectional study with a small sample size, causality between risk factors and food contamination cannot be inferred, and statistical power limited detection of differences across many individual behaviours and correction for multiple hypothesis testing. Unknown risk factors that contribute to food contamination but were not included in observations or questionnaires may remain. Ideally, samples would be taken at multiple time points throughout preparation and storage, which was logistically unfeasible, and reheating and feeding of stored foods were not observed. Future research might implement full HACCP procedures in a low‐income urban setting and establish critical limits for CCPs identified.

Self‐reported behaviour often leads to overreporting of behaviours thought to be desirable (Manun'Ebo et al., 1997). Knowledge of the study's focus on food contamination in relation to diarrhoeal risk may have produced overestimates of certain practices and biased associations with food contamination. Direct observations may have been more reliable (Curtis et al., 1993), though reactivity bias is a concern as observations only occurred once and for a short time period.

Using *Enterococcus* spp. detection in child foods to indicate faecal contamination provides no information on the diversity or prevalence of specific pathogens in food nor diarrhoeagenic viruses or parasites and may also indicate animal faecal contamination (Barnes et al., 2018). As employed elsewhere (Tsai et al., 2019), directly detecting pathogens in food, particularly those identified as important in reducing moderate‐to‐severe diarrhoea in the Global Enteric Multicenter Study (GEMS) (Kotloff et al., 2013), may improve indication of diarrhoea risk.

## 5 CONCLUSION

Despite being a small, exploratory study, our findings are consistent with existing literature suggesting that food may be an important transmission pathway for childhood diarrhoea, especially in high‐density, low‐income, urban settings with limited public infrastructure. Future research is needed to describe contamination by different food types and across different age intervals, coupled with the detection of specific diarrhoeagenic pathogens rather than generalised faecal indicator bacteria. A better understanding of the nature of foodborne risks for children living in these challenging urban environments, and how these risks might be mitigated, will support more effective diarrhoeal disease control strategies.

## CONFLICTS OF INTEREST

The authors declare that they have no conflicts of interest.

## CONTRIBUTIONS

SB, LP, and OC designed the research study. SB and LP performed the research and analysed the data. BM supported data collection. RD supported statistical analysis. SB wrote the first draft of manuscript, and all authors reviewed and contributed to subsequent drafts. All authors read and approved the final manuscript.

## Figures and Tables

**Figure D1:**
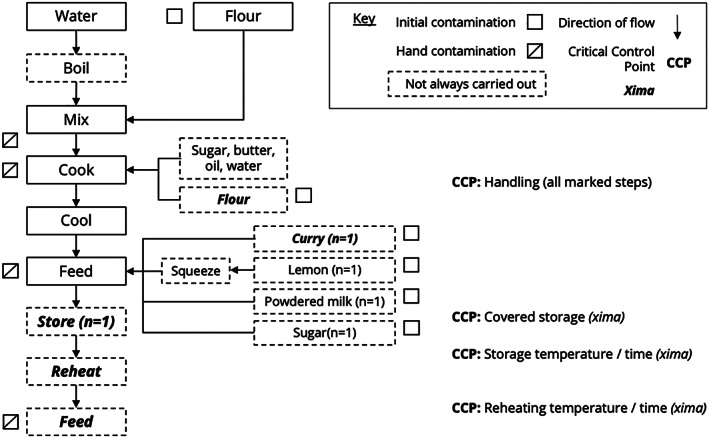
Food flow diagram for the preparation of flour‐based food (porridge and *xima*), with associated critical control points (CCPs). Steps in bold/italics are those used in the preparation of *xima*

**Figure D2:**
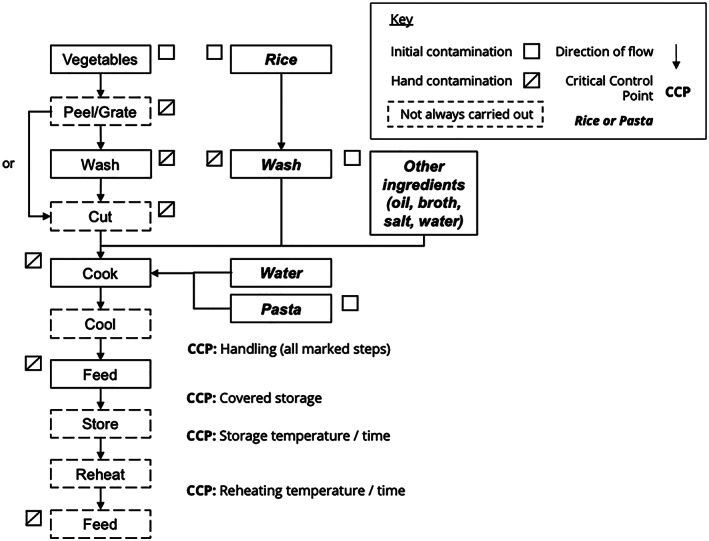
Food flow diagram for the preparation of cooked vegetables served with rice or pasta, with associated critical control points (CCPs). Steps in bold/italics are those used in the preparation of rice or pasta

**Figure D3:**
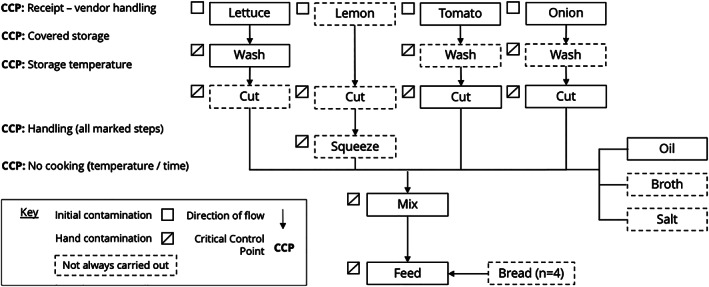
Food flow diagram for the preparation of salads, with associated critical control points (CCPs)

**Figure D4:**
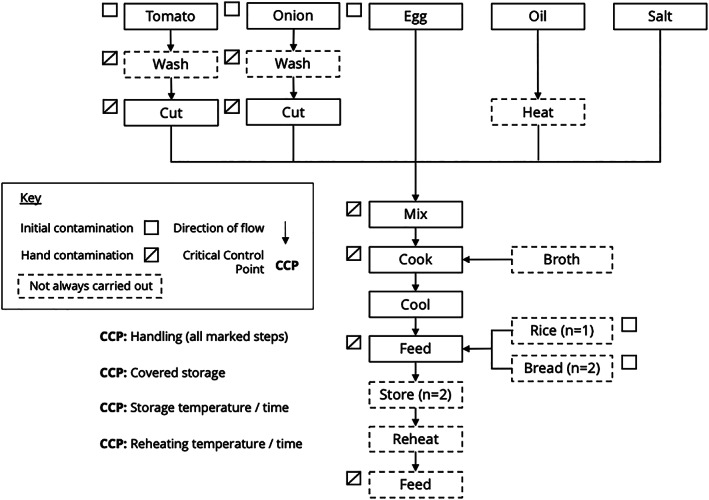
Food flow diagram for the preparation of fried egg, with associated critical control points (CCPs)

**Figure D5:**
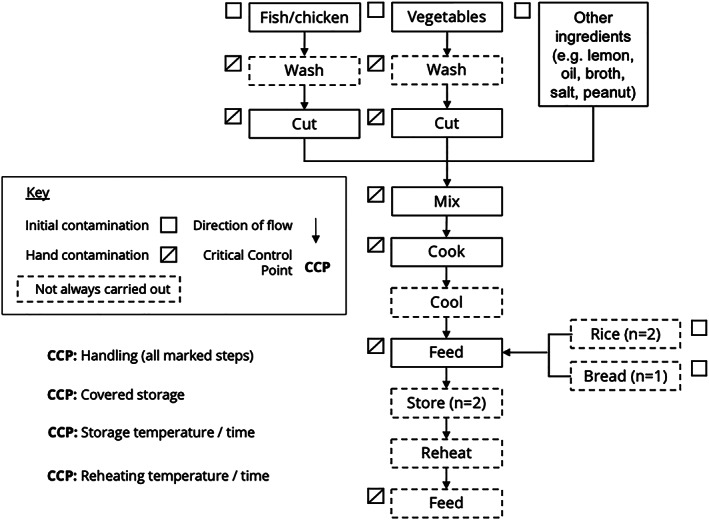
Food flow diagram for the preparation of meat/fish, with associated critical control points (CCPs)

## APPENDIX A Principal component analysis—Variables contributing to the relative wealth index

**Table A1:** Output of the principal component analysis displaying the proportion of variance of each variable explained by the first component

Variable	Proportion of variance
Household size	0.030
Crowding	−0.197
Asset ownership	
Bicycle	0.077
Cart drawn by animal	0.027
Motorcycle	0.136
Clock	0.028
Radio	0.200
Television	0.288
Mobile telephone	0.071
Refrigerator/freezer	0.179
Housing characteristics	
No. of rooms	0.195
Electricity	0.315
Grated windows	0.170
Grated door	0.102
Energy for cooking: Electricity	0.086
Energy for cooking: Charcoal	−0.005
Energy for cooking: Firewood	−0.024
Energy for cooking: Gas	0.065
Energy for cooking: Other	−0.233
Floor: Cement	0.369
Floor: Unbeaten land	−0.369
Walls: Cement	0.164
Walls: Wood or iron sheets	−0.007
Walls: Reeds/sticks/bamboo/palm	−0.297
Walls: Adobe blocks	0.034
MapSan intervention	0.127
Water source: Tap inside the house	0.043
Water source: Tap in the compound	0.232
Water source: Public tap/fountain	0.046
Water source: Neighbour's tap	−0.263
Water source: Other	0.012

*Note.* The first principal component accounts for 15% of the variance, with an eigenvalue of 4.66.

## APPENDIX B Caregiver characteristics cross‐tabulated with the binary contamination outcome

**Table B1:** Characteristic of 58 caregivers and associated frequencies, prevalence of faecal contamination (>10 colony forming units per gram) and *p* value from Fisher's exact test

Risk factor	*N* (%)	Contaminated (%)	*p* value
Caregiver sex			
Female	57 (98)	31 (54)	.47^a^
Male	1 (2)	0 (0)	
Caregiver age (years)			
<20	21 (36)	11 (52)	1.00
20–29	15 (26)	8 (53)	
30–39	14 (24)	8 (57)	
40+	8 (14)	4 (50)	
Relationship to child			
Mother	43 (74)	24 (56)	.56
Not mother	15 (26)	7 (47)	
Caregiver education			
No schooling	8 (14)	3 (38)	.70
Primary school	34 (59)	19 (56)	
Secondary or above	16 (28)	9 (56)	
Child age (months)			
6–23	18 (31)	8 (44)	.74
24–35	18 (31)	10 (56)	
36–47	13 (22)	7 (54)	
48–59	9 (16)	6 (67)	
No. of household members			
2–4	27 (47)	11 (41)	.11
5+	31 (53)	20 (65)	
No. of children under 5 years			
1	37 (64)	17 (46)	.17
2+	21 (36)	14 (67)	
Wealth quintile			
1st	12 (21)	8 (67)	.46
2nd	11 (19)	5 (45)	
3rd	12 (21)	7 (58)	
4th	11 (19)	6 (55)	
5th	12 (21)	5 (42)	
Crowding (people per room)			
<4	27 (47)	16 (59)	.44
≥4	31 (53)	15 (48)	
Water source			
Tap in compound	33 (57)	17 (52)	.80
Other	25 (43)	14 (56)	
Water stored in covered container			
Yes	49 (84)	25 (51)	.48
No	9 (16)	6 (67)	
Point of use water treatment			
Yes	10 (18)	4 (40)	.49
No	47 (82)	27 (57)	
MapSan trial arm			
Intervention	29 (50)	15 (52)	1.00
Control	29 (50)	16 (55)	
Child faeces observed on premises			
Yes	2 (3)	2 (100)	.49^a^
No	56 (97)	29 (52)	
Animals observed on premises			
Yes	39 (67)	20 (51)	.78
No	19 (33)	11 (58)	
Soap availability			
Yes	53 (91)	26 (49)	.06^a^
No	5 (9)	5 (100)	
Self‐reported HWWS			
Yes	35 (60)	16 (46)	.18
No	23 (40)	15 (65)	
HWWS after defecation			
Yes	27 (47)	12 (44)	.29
No	31 (53)	19 (61)	
HWWS after changing nappies			
Yes	3 (5)	0 (0)	.10^a^
No	55 (95)	31 (56)	
HWWS before preparing food			
Yes	22 (38)	11 (50)	.79
No	36 (62)	20 (56)	
HWWS before eating			
Yes	30 (52)	13 (43)	.12
No	28 (48)	18 (64)	
HWWS before feeding the child			
Yes	16 (28)	6 (38)	.15
No	42 (72)	25 (60)	
Washing of cooking utensils			
Yes	41 (71)	21 (51)	.77
No	17 (29)	10 (59)	
Washing of feeding utensils			
Yes	39 (67)	21 (54)	1.00
No	19 (33)	10 (52)	
Washing of food containers			
Yes	43 (74)	24 (56)	.56
No	15 (26)	7 (47)	
Washing of cooking surfaces			
Yes	7 (12)	4 (57)	1.00
No	51 (88)	27 (53)	
Utensils visibly dirty			
Yes	9 (16)	6 (67)	.48
No	28 (84)	24 (50)	
Location of cooking			
Inside	43 (74)	21 (49)	.37
Outside	15 (26)	10 (67)	
Location of feeding the child			
Inside	28 (48)	15 (54)	1.00
Outside	30 (52)	16 (53)	
Main ingredients			
Flour‐based: Porridge	11 (19)	3 (27)	.06
Cooked vegetables	27 (47)	15 (56)	
Salad	6 (10)	6 (100)	
Fried egg	5 (9)	2 (40)	
Meat/fish	9 (16)	5 (56)	
Contact with other foods (same container/utensil)			
Yes	23 (40)	15 (65)	.18
No	35 (60)	16 (46)	
Storage after cooking^b^			
Recently cooked	30 (58)	11 (37)	.09
Cooked earlier, stored	22 (42)	14 (64)	
Food stored in covered container^c^			
Yes	16 (73)	8 (50)	.05^a^
No	6 (27)	6 (100)	
Food refrigerated^c^			
Yes	1 (5)	0 (0)	.36^a^
No	21 (95)	14 (67)	
Food reheated^c^			
Yes	9 (41)	6 (67)	1.00
No	13 (59)	8 (62)	

Abbreviation: HWWS, handwashing with soap.

a Perfectly predicts the outcome.

b Out of cooked food (excluding salad).

c Out of food stored after cooking.

## APPENDIX C Bivariate negative binomial regression by risk factor

**Table C1:** Results of bivariate negative binomial regression based on enterococci counts in child foods in Maputo, Mozambique

Risk factor	Coefficient^a^	*z*	95% CI	*p* value
Caregiver sex—female (ref: Male)	(does not converge)			
Caregiver age (years)				
<20	Ref			
20–29	−0.74	−0.63	[−3.1, 1.6]	.53
30–39	−0.19	−0.16	[−2.5, 2.1]	.87
40+	−0.28	−0.14	[−4.2, 3.6]	.89
Relationship to child—Mother (ref: Not mother)	0.79	0.49	[−2.4, 4.0]	.63
Caregiver education				
No schooling	Ref			
Primary school	3.4	0.60	[−7.6, 14]	.55
Secondary or above	4.3	0.77	[−6.7, 15]	.44
Child age (months)				
6–23	Ref			
24–35	1.5	1.2	[−0.96, 4.0]	.23
36–47	0.70	0.56	[−1.7, 3.2]	.57
48–59	0.66	0.53	[−1.8, 3.1]	.59
No. of household members				
2–4	Ref			
5+	2.5	3.3	1.0, 4.0	<.01
No. of children under 5 years				
1	Ref			
2+	0.37	0.47	[−1.2, 1.9]	.64
Wealth quintile				
1st	Ref			
2nd	−2.8	−3.3	[−4.4, −1.1]	<.01
3rd	0.11	0.07	[−2.9, 3.1]	.94
4th	−0.63	−0.42	[−3.6, 2.3]	.67
5th	−0.56	−0.38	[−3.4, 2.3]	.70
Crowding (people per room)				
<4	Ref			
≥4	−0.37	−0.48	[−1.9, 1.2]	.64
Water source—Tap in compound (ref: Other)	1.1	1.2	[−0.66, 2.9]	.22
Water stored in covered container—Yes (ref: No)	−0.57	−0.22	[−5.6, 4.4]	.82
Point of use water treatment—Yes (ref: No)	−0.32	−0.29	[−2.5, 1.8]	.77
MapSan trial arm—Intervention (ref: Control)	−0.74	−0.91	[−2.3, 0.85]	.36
Child faeces observed on premises—Yes (ref: No)	2.5	0.89	[−3.0, 8.1]	.37
Animals observed on premises—Yes (ref: No)	0.07	0.11	[−1.2, 1.4]	.91
Soap availability—Yes (ref: No)	−0.16	−0.09	[−3.4, 3.1]	.93
Self‐reported HWWS—Yes (ref: No)	0.61	0.60	[−1.4, 2.6]	.55
HWWS after defecation—Yes (ref: No)	−0.35	−0.45	[−1.9, 1.2]	.65
HWWS after changing nappies—Yes (ref: No)	−26	−12	[−30, −21]	<.01
HWWS before preparing food—Yes (ref: No)	1.3	1.5	[−0.4, 2.9]	.14
HWWS before eating—Yes (ref: No)	0.94	1.1	[−0.74, 2.6]	.27
HWWS before feeding the child—Yes (ref: No)	−0.52	−0.45	[−2.8, 1.8]	.65
Washing of cooking utensils—Yes (ref: No)	1.2	1.0	[−1.1, 3.5]	.32
Washing of feeding utensils—Yes (ref: No)	1.0	0.99	[−0.98, 3.0]	.32
Washing of food containers—Yes (ref: No)	1.1	0.71	[−1.9, 4.0]	.48
Washing of cooking surfaces—Yes (ref: No)	−0.04	−0.01	[−5.7, 5.6]	.99
Utensils visibly dirty—Yes (ref: No)	0.17	0.15	[−2.0, 2.4]	.88
Location of cooking—Outside (ref: Inside)	−0.07	−0.10	[−1.5, 1.4]	.92
Location of feeding the child—Outside (ref: Inside)	0.73	0.98	[−0.73, 2.2]	.33
Main ingredients (ref: Ingredient not included)				
Flour‐based: Porridge	−4.2	−5.5	[−5.7, −2.7]	<.01
Cooked vegetables	1.2	1.5	[−0.32, 2.7]	.12
Salad	0.19	0.17	[−2.0, 2.4]	.87
Fried egg	−3.4	−0.43	[−19, 12]	.67
Meat/fish	−0.20	−0.14	[−3.1, 2.7]	.89
Contact with other foods – Yes (ref: No)	0.47	0.56	[−1.2, 2.1]	.57
Food stored after cooking (ref: Food cooked and not stored)	1.3	1.7	[−0.18, 2.8]	.09
Food stored in covered container (ref: Stored uncovered)	−0.38	−0.38	[−2.4, 1.6]	.71
Food refrigerated (ref: Stored at ambient temperature)	−27	−19	[−30, −25]	<.01
Food reheated (ref: Stored and not reheated)	1.3	1.1	[−0.99, 3.6]	.27

*Note.* Hygiene risk factors are based on self‐report.

Abbreviations: CI, confidence interval; HWWS, handwashing with soap.

a Coefficient relates to the increase in log count.
